# Characterizing T1 in the fetal brain and placenta over gestational age at 0.55T

**DOI:** 10.1002/mrm.30193

**Published:** 2024-07-05

**Authors:** Jordina Aviles Verdera, Raphael Tomi-Tricot, Lisa Story, Mary A. Rutherford, Sebastien Ourselin, Joseph V. Hajnal, Shaihan J. Malik, Jana Hutter

**Affiliations:** 1 Centre for the Developing Brain, School of Biomedical Engineering and Imaging Sciences, King’s College London, London, UK; 2 Biomedical Engineering Department, School of Biomedical Engineering and Imaging Sciences, King’s College London, London, UK; 3 MR Research Collaborations, Siemens Healthcare Limited, Camberley, UK; 4 Women’s Health, GSTT, London, UK; 5 Smart Imaging Lab, Radiological Institute, University Hospital Erlangen, Erlangen, Germany

**Keywords:** fetal MRI, low cost, low-field, relaxometry

## Abstract

**Purpose:**

T1 mapping and T1-weighted contrasts have a complimentary but currently under utilized role in fetal MRI. Emerging clinical low field scanners are ideally suited for fetal T1 mapping. The advantages are lower T1 values which results in higher efficiency and reduced field inhomogeneities resulting in a decreased requirement for specialist tools. In addition the increased bore size associated with low field scanners provides improved patient comfort and accessibility. This study aims to demonstrate the feasibility of fetal brain T1 mapping at 0.55T.

**Methods:**

An efficient slice-shuffling inversion-recovery echo-planar imaging (EPI)-based T1-mapping and postprocessing was demonstrated for the fetal brain at 0.55T in a cohort of 38 fetal MRI scans. Robustness analysis was performed and placental measurements were taken for validation.

**Results:**

High-quality T1 maps allowing the investigation of subregions in the brain were obtained and significant correlation with gestational age was demonstrated for fetal brain T1 maps (*p <* 0.05) as well as regions-of-interest in the deep gray matter and white matter.

**Conclusions:**

Efficient, quantitative T1 mapping in the fetal brain was demonstrated on a clinical 0.55T MRI scanner, providing foundations for both future research and clinical applications including low-field specific T1-weighted acquisitions.

## 1 Introduction

Fetal MRI is a rapidly evolving and exciting field of research facilitating insights into early human development in vivo. It has a complementary role to ultrasound in the antenatal diagnosis of fetal and placental anomalies due to its high resolution and excellent soft tissue contrast. Assessment of the fetal brain is currently the most common indication for both clinical and research fetal MR scans. However, recent advances have now facilitated evaluation of additional fetal organs in both the thorax and abdomen, the placenta as well as surrounding maternal tissues.

Historically most fetal MRI examinations have been performed at 1.5 or 3T, but the increasing availability of lower field strength scanners and their distinct advantages for fetal imaging have contributed to renewed interest in low field imaging for the assessment of of the fetus.^1–3^ Artifacts commonly encountered in fetal MRI include geometric distortion originating from air–tissue interfaces near the maternal bowels and B1-artifacts related to the high conductivity of amniotic fluid. These artifacts are reduced at low field compared to higher field strengths due to increased B0-homogeneity and B1-homogeneity. Low field scanners are also associated with an increased bore size, with maintained field homogeneity, which facilitates imaging of larger patients. This is increasingly relevant due to rising obesity rates internationally, with over 25% of all pregnancies in the United States and United Kingdom characterized as obese in 2020.^4^ This is even more pertinent due to the challenges of performing and obtaining reliable measurements from ultrasound examinations in obese women, a population which is at increased risk of pregnancy complications such as pre-eclampsia and gestational diabetes, and hence in whom accurate evaluation is essential.

Both anatomical and functional techniques are widely utilised in fetal MRI. T2-weighted imaging is the most common technique for anatomical assessment due to the higher water content within structures such as the white matter in the brain while T1 imaging has played a less-prominent role to date. However, it can provide enhanced evaluation of hemorrhagic lesions and calcifications within cerebral tissue^5^ and has particular utility for assessment of the pituitary gland, midline brain defects, and myelination status over gestation.^5^

Quantitative and functional techniques have shown potential for providing additional information about tissue properties, physiological processes, and organ function. These include T2* relaxometry,^6^ which provides an indirect assessment of oxygen content of maternal and fetal organs and diffusion MRI, facilitating assessment of tissue microstructure for example in the development of cerebral white matter.^7^ Quantitative T1 mapping has also been performed in a limited number of studies in the fetal brain at 3^8^ and 1.5T^9^ using Look-Locker inversion recovery and a multidynamic multi-echo technique. However, the vast majority of quantitative fetal T1 measurements performed to date have focused on the placenta. These were performed across different field strengths, including 0.5T on a dedicated EPI scanner,^1,10^ clinical 1.5T scanners^11–14^ and 3T scanners.^15,16^ A variety of imaging techniques were employed, including inversion recovery type sequences,^1,12,14,16^ Look-Locker sequences,^1^ single-shot fast spin echo sequences,^11^ multiple flip angle FFE sequences^13^ and Fingerprinting MRI.^15^ Results of these studies also varied, reporting either a decrease in placental T1 or no significant correlation with gestational age in healthy control subjects as well as reduced values in small-for-gestational-age,^10^ fetal growth restriction^10,11,14^ or pre-eclampsia.^10^ Extension of T1 mapping to the fetal lungs^17^ was also demonstrated. Echo planar imaging (EPI) is widely used for quantitative fetal MRI such as T1 mapping due to the intrinsic ability to freeze fetal motion within the slices and the low number of radiofrequency pulses and hence reduced radiofrequency heating. Furthermore, in the adult brain, slice-shuffling as a technique to efficiently acquire whole-brain T1 values has been proposed but this has not previously been utilized in the context of fetal imaging.^18^

**Figure 1:**
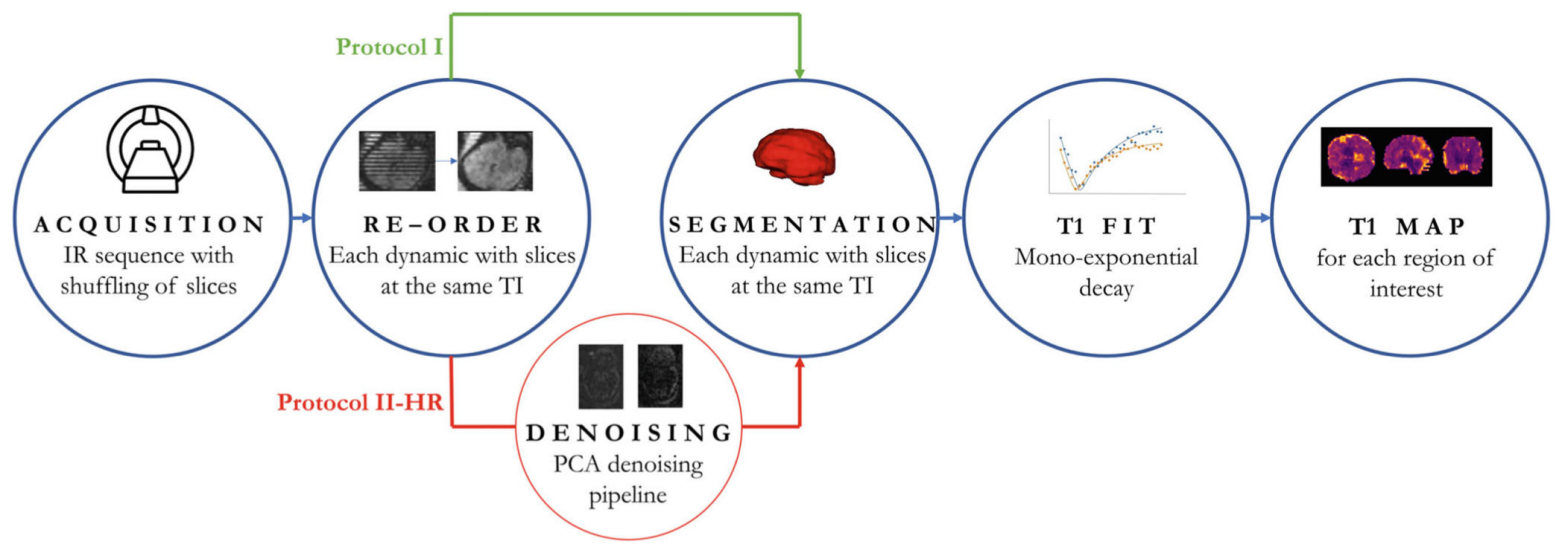
Processing pipeline from acquisition to T1 map.

This study aims to comprehensively map T1 values across gestation in the fetal brain on commercially available 0.55T MRI scanners (Figure 1). It aims to address the paucity of fetal brain T1 values currently available and to provide a key step toward quantitative fetal relaxometry. In addition, to allow comparison with the literature mainly focusing on the placenta, placental T1 is studied over gestational age. It builds on the slice-shuffling proposed by Ordidge et al.^18^ and exploits several properties of low-field MRI. Specifically, the reduced T1 allows more efficient acquisitions as well as increased homogeneity whereas the increased T2* enables EPI readouts with reduced artifacts and a more favorable signal evolution.

## 2 Methods

### 2.1 Patient selection and preparation

Women were recruited to an ethically approved study (MEERKAT, Dulwich Ethics Committee, December 8, 2021) from antenatal clinics, antenatal wards and self-referral between May 2022 and May 2023. Exclusion criteria included: maternal age <16 years and >55 years, contraindications for MRI such as metal implants, multiple pregnancies and weight >200 kg. Women with both uncomplicated pregnancies imaged as healthy volunteers and those referred for clinical imaging were included.

Data was acquired on a clinical 0.55T system (MAGNETOM Free.Max, Siemens Healthcare) using a blanket-like six-element coil (BioMatrix Contour L Coil, Siemens Healthcare) and a permanent nine-element spine coil built into the scanner bore. Women were scanned in a head-first supine position with extra head, back and leg rests as required. The bore diameter is 80 cm, with a distance of 50 cm from the patient table to the upper inner-bore surface, and the length of the system from cover to cover is 165 cm. Continuous heart rate and intermittent blood pressure monitoring were undertaken together with frequent verbal interaction. A break was offered half-way through the examination, but declined by most participants.

### 2.2 Slice-shuffled inversion-recovery sequence

**Figure 2:**
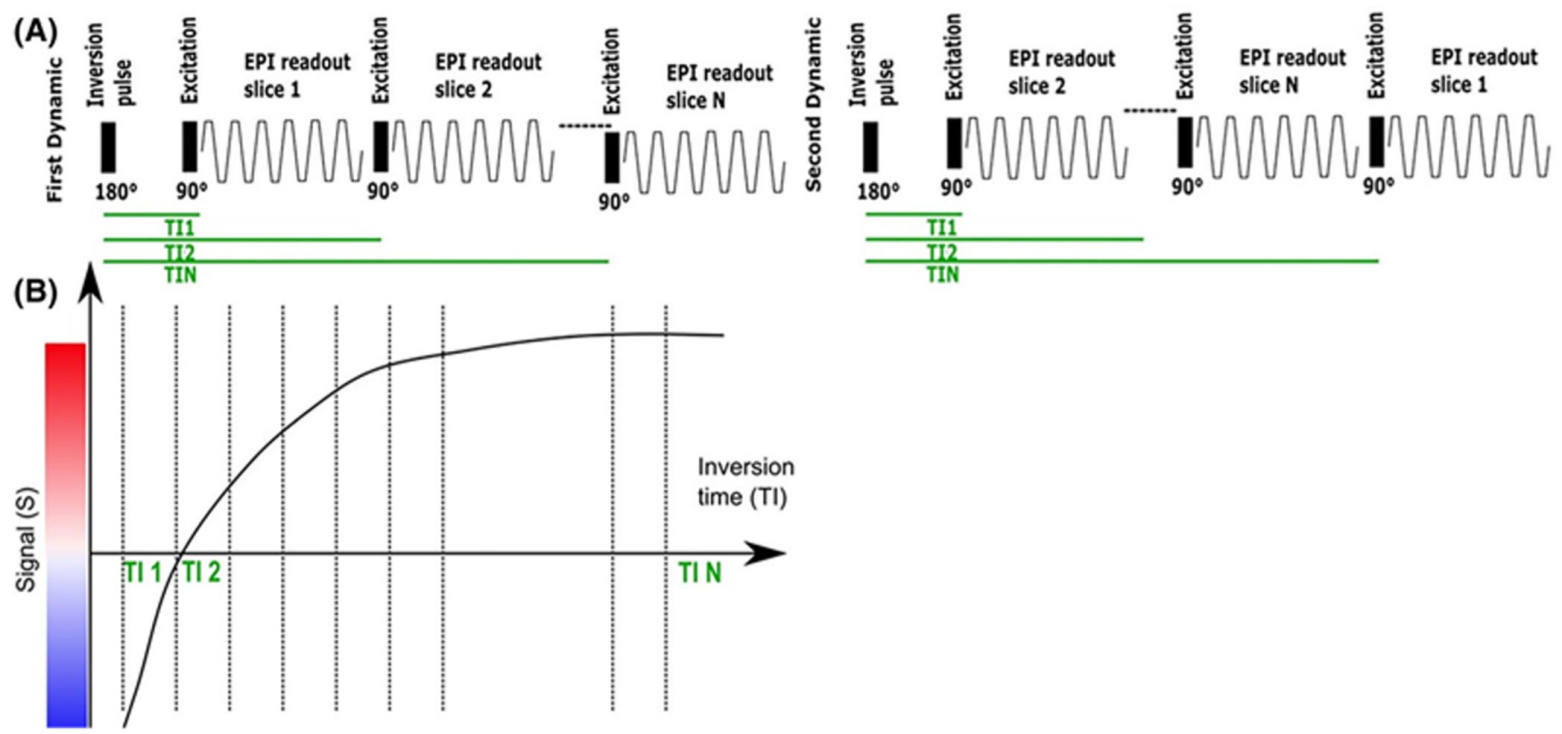
Pulse Diagram for the T1 sequences employed. (A) Inversion-recovery slice-shuffled single-shot Gradient Echo EPI with a nonselective adiabatic pulse. (B) The signal recovery curve illustrated over subsequent excitations.

A clinical gradient echo single-shot EPI sequence was modified to include a global nonselective adiabatic inversion pulse and shuffling of the *N*_*s*_ slices, previously reported in the adult brain,^18,19^ to optimize the acquisition efficiency (see Figure 2A). Each slice in the TR had a different ascending inversion time with *N* = *N*_*s*_ total inversion times being sampled (see Figure 2B). To achieve complete mapping of each inversion time (TI) for each slice, in each subsequent dynamic moving the geometrical location of the first acquired slice in time by one slice position resulting in a complete acquisition after *N*_*d*_ = *N*_*s*_ dynamics.

### 2.3 Experiments

Fetal brain quantitative T1 datasets for this study were acquired with the described sequence (See Table 1 for all parameters). Data sets were acquired in axial orientation to the fetal brain to facilitate postprocessing and visualization. In addition, to evaluate robustness, the data were acquired twice in a total of seven cases, with the repetitions spaced out at least 20 min without maternal repositioning.

Placental imaging for validation purposes was performed using a lower-resolution protocol (see Table 1 for all parameters). A thick section of the placenta was acquired in an orientation axial to the mother to achieve maximal robustness to maternal breathing motion.

### 2.4 Analysis

**Figure 3:**
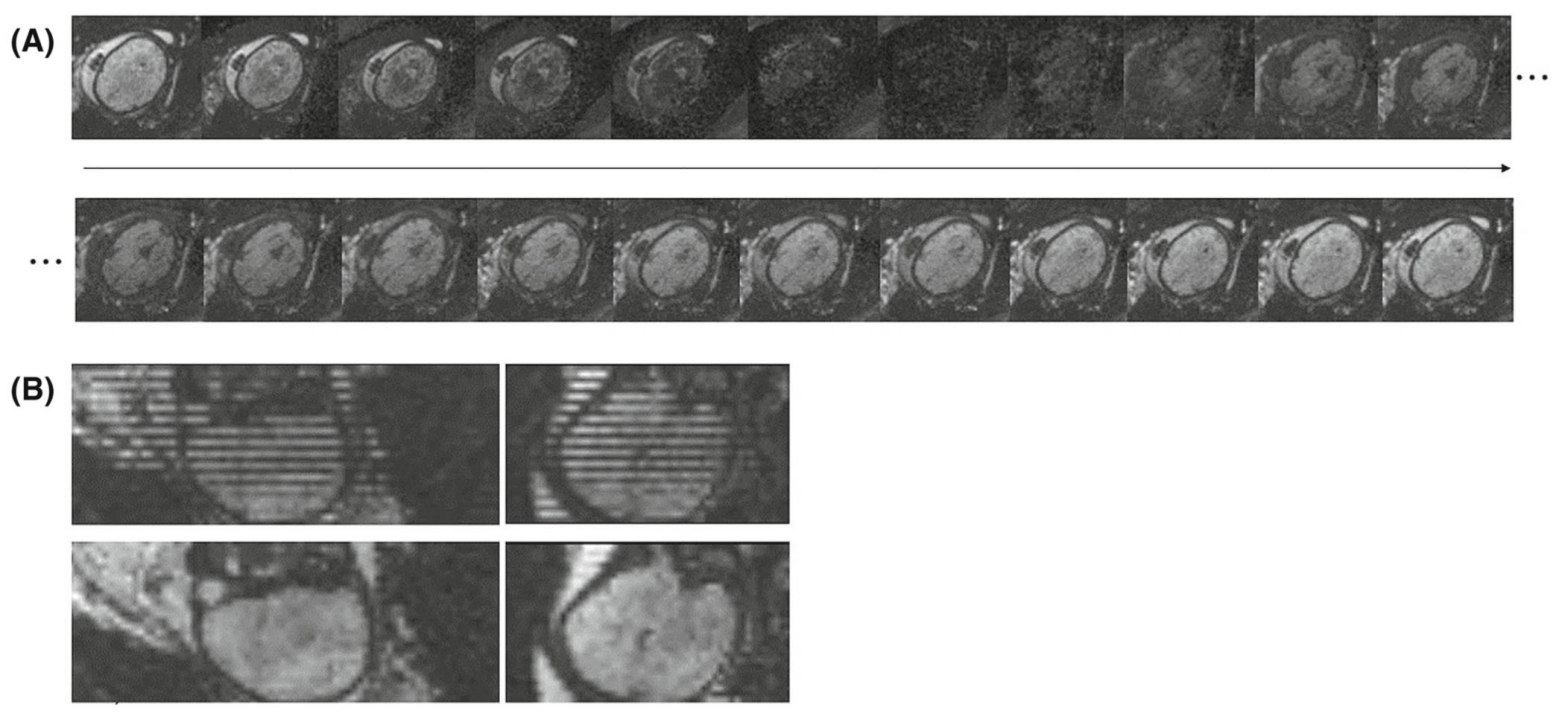
Example at 32 + 6 weeks GA showing in (A) the contrasts obtained for an axial slice over different inversion times (TIs) (left to right and top to bottom) as well as in (B) the coronal and sagittal views of the first dynamic each slice at a different TI (top) and same views with the reordered slices, resulting in all slices at same TI (bottom).

In the resulting dynamics every slice has a different TI. Resorting along all dynamics results in volumes with consistent TI. Figure 3 shows (A) a graphical representation of the dynamics over time, clearly depicting the individual contrasts in the same slice over time and (B) the stripping pattern due to the shuffling (top) together with the reordered data (bottom). Next, a visual motion assessment was performed to discard cases with extreme motion that would compromise quality of the calculated T1 maps.

Denoising using Principal Component Analysis implemented in MRtrix3 was applied.^20–22^ Noise level estimation and denoising steps were performed in the complex image to reduce Riccian bias.^21^ Finally, the magnitude data was fit to a mono-exponential decay function using least squares optimization (Equation 1), where T1 is expressed in ms with bounds [0, 5000], K the dimensionless inversion efficiency with bounds [0, 1], TIs are the inversion times and *M*_0_ the proton density with bounds [0, 1000].

(1) $$S=M_{0}\;abs\left(1-2\;\text{K}\;e^{-\frac{{\text{TI}}_{i}}{\text{T1}}}\right).$$

Finally, the whole brain volume was segmented manually in mrview^22^ by one researcher with 9 years of experience in fetal MRI, carefully avoiding any maternal structures and amniotic fluid. In addition, regions-of-interest slices were chosen in the white matter, focusing on the hemispheric white matter in the centrum semiovale area and deep grey matter, focusing on the region around the thalamus. Linear regression analysis was performed in the control population using statsmodel in python, resulting in the intercept, slope, confidence intervals, *p*-values and R2 values for the whole brain as well as the brain white matter (WM) and gray matter (GM) regions. The repeated acquisitions were analyzed using the same steps with independent masking performed and analyzed using Bland–Altman plots.

The placenta data was processed similarly in the described way by manual segmenting and fitting to a mono-exponential decay function. No filtering was performed.

## 3 Results

Complete datasets, including complex data at all steps of the analysis, and analysis code are available to interested academics upon request.

### 3.1 Brain

Fetal brain quantitative T1 data was acquired in 38 women (among these seven cases had repeated acquisition), with gestational ages ranging from 25.2 to 38.4 weeks (mean 32 weeks), BMI ranging from 21.8 to 47 kg/m^2^ (mean 29.4 kg/m^2^) and maternal age 20–45 years (mean 33 years), all representative of the local population and displayed graphically in Figure S1.

**Figure 4:**
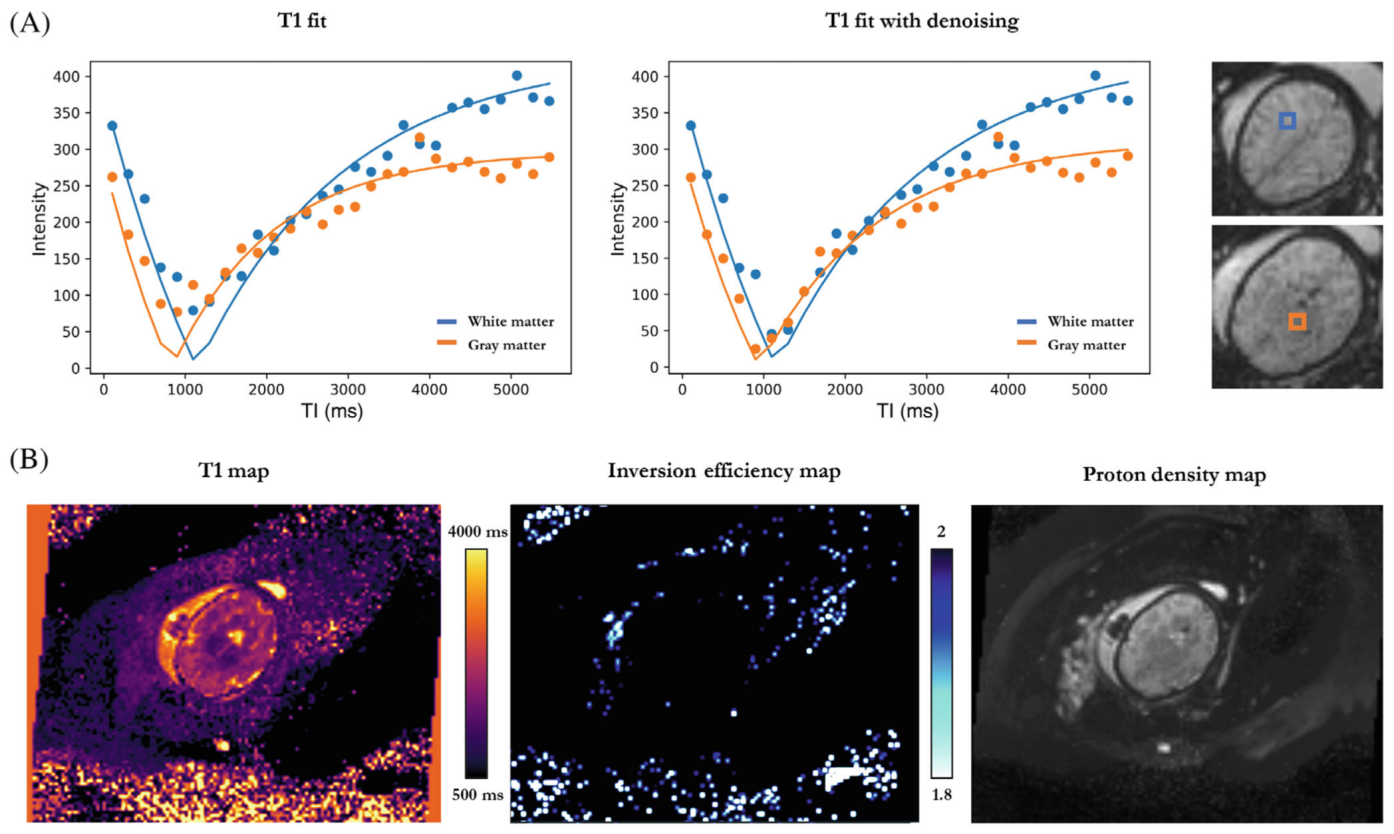
T1 fetal brain results for a subject at 32 + 6 weeks GA. (A) Individual data points across inversion time and their corresponding T1 fits for hemispheric white matter (blue) and deep grey matter (orange), without and with the denoising step. Selected voxels are shown in a colored box next to the plots. (B) Axial view of the whole image T1, inversion efficiency and proton density maps, from left to right.

High-quality T1 brain maps were obtained for a total of 35 subjects with three discarded due to severe motion. Examples of the discarded cases are illustrated in Figure S2. Figure 4 shows (A) individual data points across all TIs with their corresponding T1 fits for two different voxels in the white and grey matter, as well as (B) the whole image T1 map, proton density (PD) map and inversion efficiency (IE) map for the same fetal brain acquisition. Mean Inversion efficiency for all included cases was 1.98 ± 0.013.

**Figure 5:**
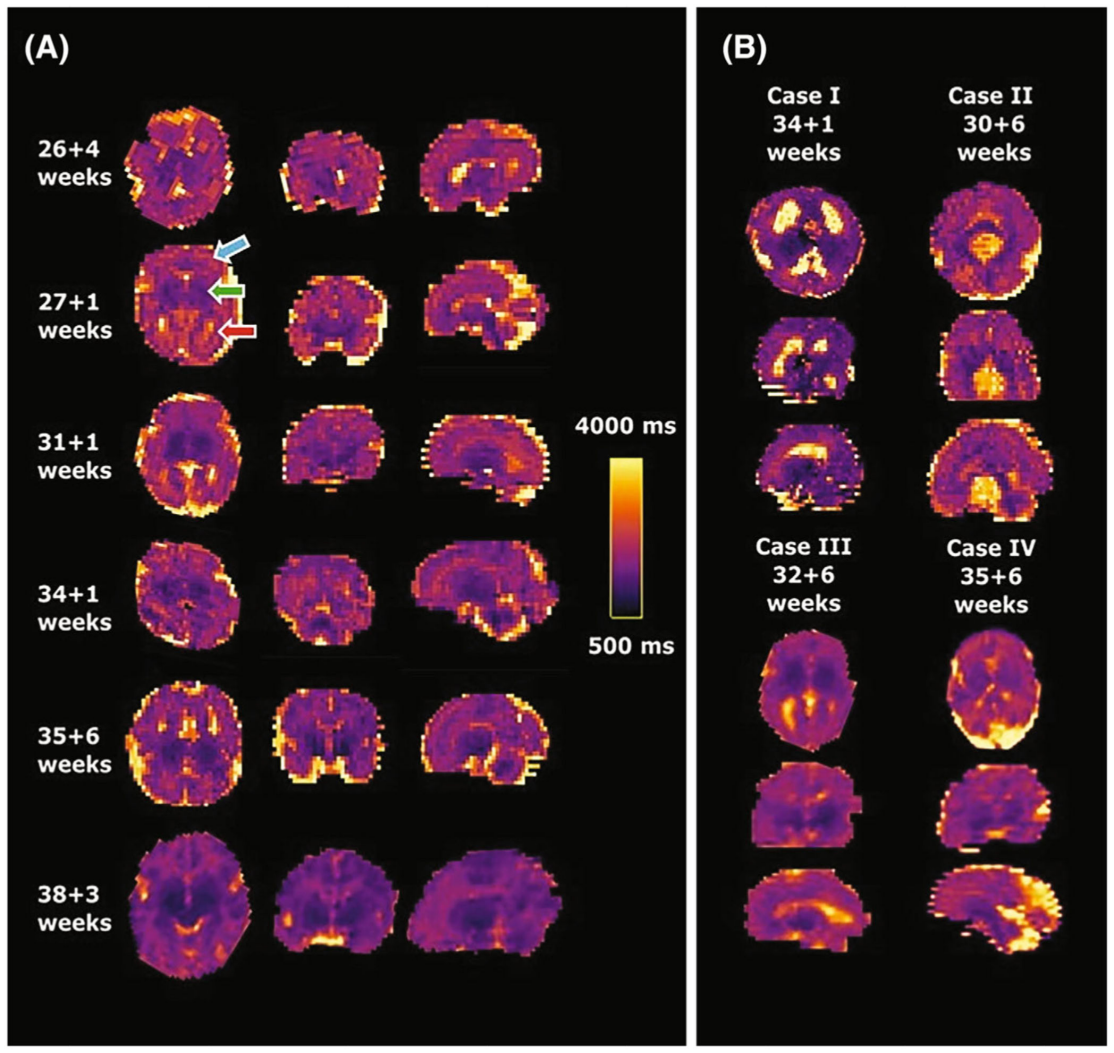
Obtained high-resolution brain T1 maps in axial, coronal and sagittal orientation from (A) six control fetuses sorted by gestational age and (B) four clinical examples with ventriculomegaly, mid-line brain cyst, unilateral ventriculomegaly and enlarged cisterna magna from left to right and top to bottom are shown. The same scaling is used for all cases as indicated in (A). Most cases were acquired axial to the fetal brain, these acquired in tilted orientations were rotated to true radiological planes. Blue arrow points at white matter, green arrow to deep grey matter and red arrow to ventricles.

**Figure 6:**
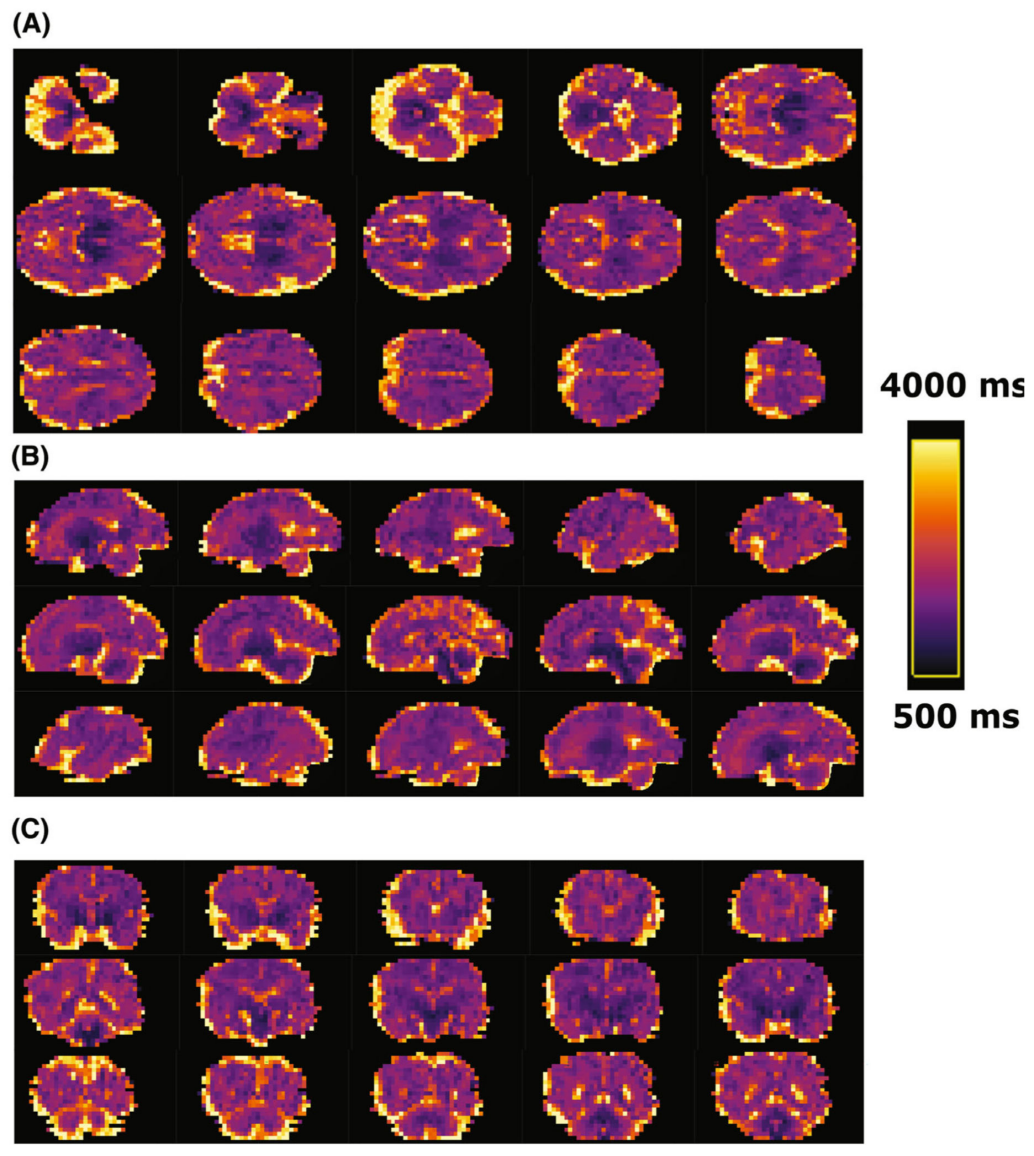
(A) Axial, (B) sagittal, and (C) coronal views from subsequent mid-brain slices in a brain T1 map from a healthy fetus images at 35 + 6 weeks gestational age using the high-resolution protocol. (A) Axial slices from ventral to dorsal; (B) Sagittal slices from left to right; (C) coronal from rostral to caudal.

Figure 5A shows results from six healthy cases across different gestational ages. Clearly distinguishable regions of the brain can be appreciated from an early gestational age. The clinical cases in Figure 5B display the increased size of both (top left) or one ventricle (bottom left), the mid-line cyst (top right) and the dilated cisterna magna (bottom right) clearly. Figure 6 displays multiple slices in all three directions for a case at 35 + 6 weeks gestational age.

**Figure 7:**
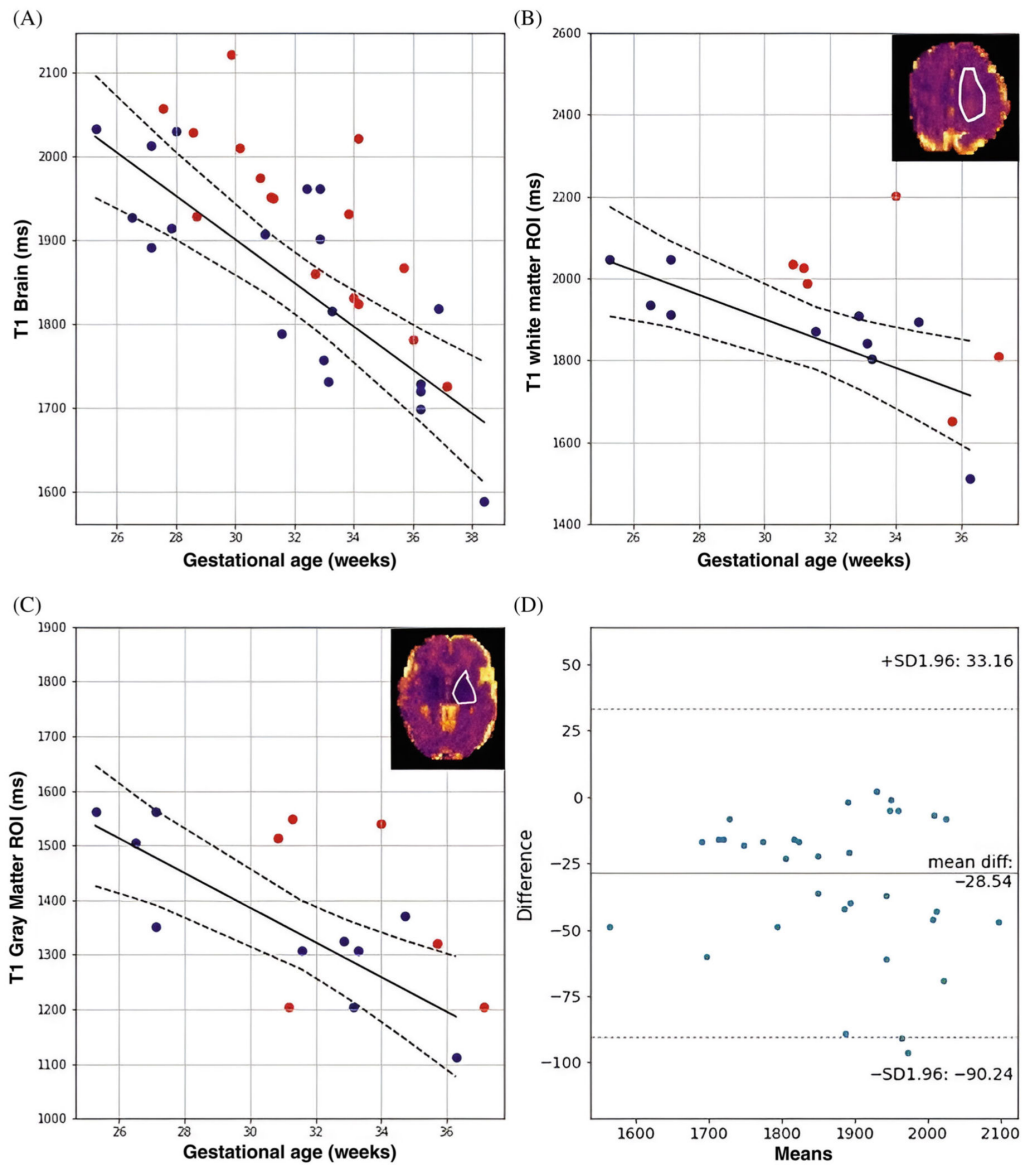
Mean quantitative T1 results for (A) the whole brain and the selected brain regions (B,C) over gestational age. (B,C) also illustrate these selected regions in the upper right corner with a white outline. The blue dots denote the values from the control participants, red dots from clinical cases with fetal brain-related pathologies, the black lines are the regression line and dotted lines are the confidence interval for control participants. Finally, (D) A Bland–Altman plot for mean T1 for the fetal brain before and after the denoising step is given.

The quantitative results in Figure 7A show significant trends over gestational age for the control cases for whole-brain mean T1 values with a decline of 25 ms/week for the whole brain data (*p <* 0.005). The ROI-specific plots in Figure 7B,C highlight significant correlation in the GM (*p <* 0.005, decay of 31 ms/week) and WM (*p <* 0.05, decay of 29 ms/week). The Bland–Altman plot in Figure 7D for the fetal brain data before and after the denoising postprocessing step shows small difference of 28.54 ms in mean T1, slightly reduced after filtering. Finally, Figure S4 shows T1 results displayed with three colors for three chosen BMI ranges and Figure S5 the robustness results for the seven cases with repeated acquisition.

### 3.2 Placenta

All acquired placenta data sets showed only moderate motion and could be analyzed. Placental datasets over gestation are shown in Figure S3A,B and quantitative results over gestation in Figure S3C showing a statistically significant decline of 6 ms/week (*p <* 0.005).

## 4 Discussion

### Brain

We have demonstrated feasibility of efficient T1 mapping in the fetal brain on commercial 0.55T MRI scanners. Previous literature regarding T1 values in the fetal brain is limited. However, our finding of a significant decay over GA in fetal WM is in accordance with a previous fetal study which included brain WM ROIs.^9^ The observed decrease in GM, including the decay over GA, are also aligned with published neonatal brain T1 relaxation times.^23^ The shortening of T1 in white matter over gestational age can be explained by the known decrease of free water, which is bound with increasing strength to myelin constituents such as glycolipids and cholesterol, and also the reduction of the water rich extracellular matrix with increasing gestational age. The latter is particularly essential at earlier gestations to facilitate axonal growth and development.^24^ The decay of T1 in GM over GA may also be explained by the tissue being richer in nuclei with a similar decay in the water-rich extracellular matrix.

### 4.1 Placenta

Placental quantitative T1 measurements were obtained for validation purposes in this study. Trends were in accordance with previously published literature at both 0.55T^10^ and at higher field strengths (1.5 and 3T).^14,16,25^ The absolute values were, however, on average lower (1320 ms at 20 weeks and 1150 ms at 40 weeks)^14,16,25^ which is in line with the expected changes associated with reduced field strength. Importantly, the values found in this study were similar to previously reported T1 values at 0.5T,^10^ albeit slightly lower on average. This reduction may be attributable to the different resolutions used between the two studies. Gowland et al. used a larger voxel size which may have resulted in partial volume effects from the higher signal intensity close to the maternal basal plate. The placental reduction in T1 was previously associated with the changes in fetal hematocrit over gestation^26,27^ and can also be linked to the increasing density of the villi in the placental parenchyma.

### 4.2 Strengths and limitations

We have demonstrated the ability to obtain high-quality quantitative results on a novel clinical low field scanner using a modified but efficient sequence. Its robustness was illustrated with repeated acquisitions resulting in a good agreement without bias related to gestational age. Including women with a BMI of over 40 kg/m^2^ further demonstrates the feasibility to obtain high-quality data even in this challenging cohort. We have quantified T1 not only in the whole brain but also within subregions and have showed significant correlation between T1 values and gestational age. We have also demonstrated the ability of the methodology to achieve a resolution fine enough to assess smaller areas within the cerebral tissue on a low field scanner. While low field fetal MRI is not yet widely available, the benefits we have reported and the recent fast growing interest in the area means that this work has importance in demonstrating usability and robustness.

The voxel analysis of individual data points across all TIs and their corresponding fits in all cases (example in Figure 4) demonstrate the importance of adding the denoising step in the post-processing pipeline to tackle decreased SNR due to increased resolution. As shown in Figure 4, once denoising is applied, the data follows a typical recovery curve.

A number of limitations should be noted with regards to this work. This includes the fact that no advanced motion correction was performed which, together with the shuffling of the slices from *N*_*s*_ different volumes acquired over *N*_*s*_ TIs, resulted in significant inconsistencies within the TI-sorted volumes in cases of extreme motion. Although this only occurred in three cases for this study, these datasets were discarded. In order to address this, future studies could utilise slice-to-volume tools to align the slices within the TI volumes. The shuffling approach used also needs special consideration with regards to how different contrasts are handled; acquiring multiple dynamics and resorting to the TIs before reconstruction may be required. Furthermore, to increase efficiency, as well as to enable for example whole-placental coverage, a simultaneous multislice (SMS) acquisition with acceleration factor of 2 could be employed to cover the same spatial extent of *N*_*s*_ slices with only *N*_*s*_/2 excitations, meaning two slices share the same TI in each TR. The total number of different TIs and thus required dynamics to achieve full sampling would be decreased to *N* = *N*_*s*_/2. This gain in efficiency would be especially well suited to the reduced T1 at low field. In addition, the time-locked three-dimensional information for the slices acquired simultaneously could help with fetal motion tracking and correction. Furthermore, only magnitude data was used in the fitting process. However, the use of complex data in that step would allow to polarity restore the employed equation, thus making the fitting more efficient. It should also be noted that, the cohort composed of both healthy pregnancies and 16 clinical cases is heterogeneous. Future studies are required to explore individual pathologies in detail to provide more insight into clinical utility.

Beyond the brain, T1 mapping allows differentiation of tissues with short T1 times such as meconium, fat, liver, thyroid, thymus or pituitary glands which may have utility in the context of fetal infection.^28^ Diagnostic value has also been demonstrated for gastrointestinal anomalies and abdominal wall defects, due to the ability to visualize the paramagnetic substrates such as, magnesium, iron and manganese (found in meconium)^29,30^ and to analyze fetal fat deposits.^31^ However, no fetal body T1 mapping was performed in these previously reported studies, hence its utility should be explored in future work with regards to the endocrine system and gastrointestinal tract.

While the shorter T1 times are a benefit in the context of efficient T1 mapping, they pose significant challenges for example Arterial Spin Labeling and Time-of-Flight techniques.

### 4.3 Implications and next steps

The results demonstrate a clear and significant change of fetal brain T1 values over gestational age and the ability of the low field system to quantify these objectively. The resulting normal curves over gestational age as well as the detailed values obtained for relevant brain regions of interest could provide the foundation to develop T1-weighted sequences and contrasts specifically adapted to fetal MRI at 0.55T and exploiting its properties and ability to offer bespoke visualization and reporting for pathology. T1 mapping targets different tissue properties compared to the widely performed T2* mapping and can hence play an important complementary role.

In-line with recent research in women diagnosed with fetal growth restriction or pre-eclampsia, further steps could also include scanning a cohort of such high-risk women to gain further insight into the progression of placental T1 in these diseases, potentially resulting in earlier diagnoses and providing a means of monitoring disease progression or future therapies. Furthermore, building upon the work demonstrating a role for T1-weighted imaging in gastrointestinal malformations and in mapping fetal abdominal fat further studies will explore its utility in other fetal organs.

## 5 Conclusion

Efficient and fast fetal T1 mapping has been demonstrated in a cohort of 35 women on a commercially available low field (0.55T) MR scanner. The characterization of T1 in different fetal brain regions paves the way for further development of bespoke contrasts and the exploration of T1-weighted imaging at this field strength. The larger bore size, limited susceptibility artifacts and more homogeneous B1 field widen access to fetal MRI for previously excluded groups, such as the increasing number of women with high BMI who are currently under-served by conventional antenatal imaging techniques.

## Supplementary Material

Supporting Information

## Figures and Tables

**TABLE 1:** Considered protocols.

Protocol ID	Protocol parameters	Data sets
Brain	400 × 400 mm^2^, 25 slices, Resolution 3 × 3 × 3.1 mm^3^ TE = 104 ms, TR = 5000 ms, No acceleration, No PF, TA = 2:25 TI = [104, 304, 501, 701, 899, 1099, 1296, 1496, 1694, 1894, 2091, 2291, 2489 2689, 2886, 3086, 3284, 3481, 3681, 3879, 4079, 4276, 4476, 4674, 4874]	38
Placenta	FOV = 400 × 400 mm^2^, 25 slices, Resolution 4 × 4 × 4 mm^3^ TE = 80 ms, TR = 3820 ms, No acceleration, No PF, TA = 1:51 TI = [80, 232, 385, 535, 687, 840, 992, 1142, 1295, 1447, 1600, 1750, 1902 2055, 2207, 2357, 2510, 2662, 2815, 2965, 3117, 3270, 3422, 3572, 3725]	34
