# Supplementary material for: Characterizing T1 in the fetal brain and placenta over gestational age at 0.55T
Source: Magn Reson Med. Author manuscript; Available in PMC 2024 Dec 16. (PMC7617244; doi:10.1002/mrm.30193)
Supplement: Supporting Information [file EMS201789-supplement-Supporting_Information.pdf]

Supplementary material

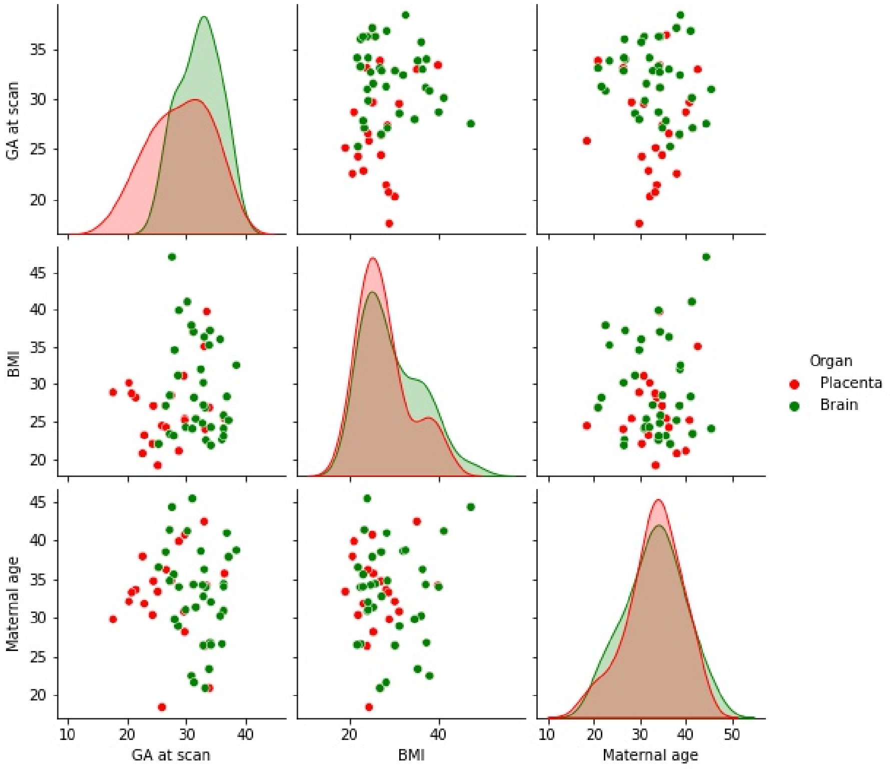

Fig. S1. Demographics of the studied cohort.

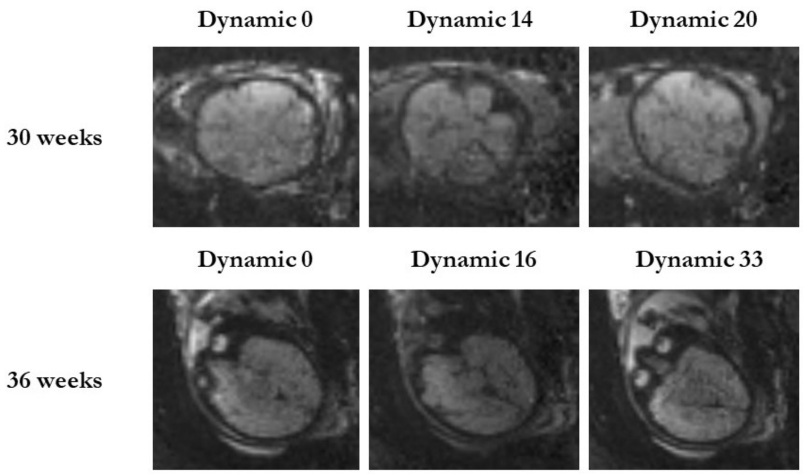

Fig. S2. Two examples of the discarded cases due to motion across different dynamics (different Inversion Time (TI)) showing change in fetal brain position affecting quality of the T1 maps.

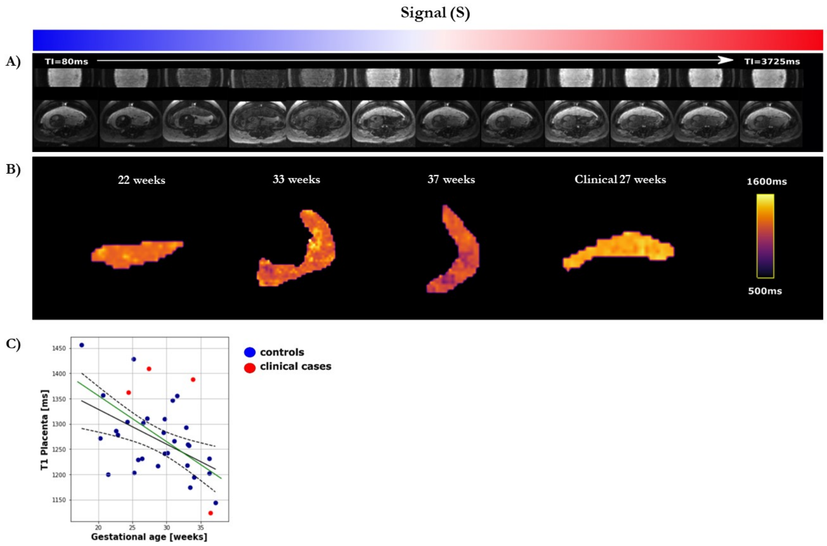

Fig. S3. Exemplar placental data and T1 maps. A) Reformatted coronal (top row) and transverse placental slice (bottom row) over a subset of the considered 28 inversion times. The color bar on top illustrated the signal recovery matched to the schemata in Figure 2. B) Transversal T1 maps of three control cases over gestation and one clinical case with type 1 diabetes, depicting the lobular structure of the placenta. (C) Quantitative placenta mean T1 over gestational age. The green line corresponds to the previous data from Gowland at al at 0.5T.

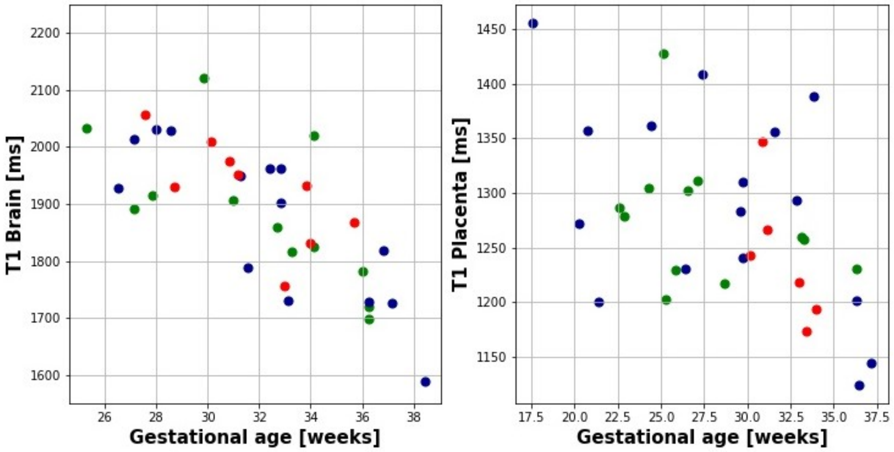

Fig. S4. Quantitative results colored by BMI. Green colored dots denote low BMI (<25), blue normal BMI (>25-<35) and high BMI (>35) in red.

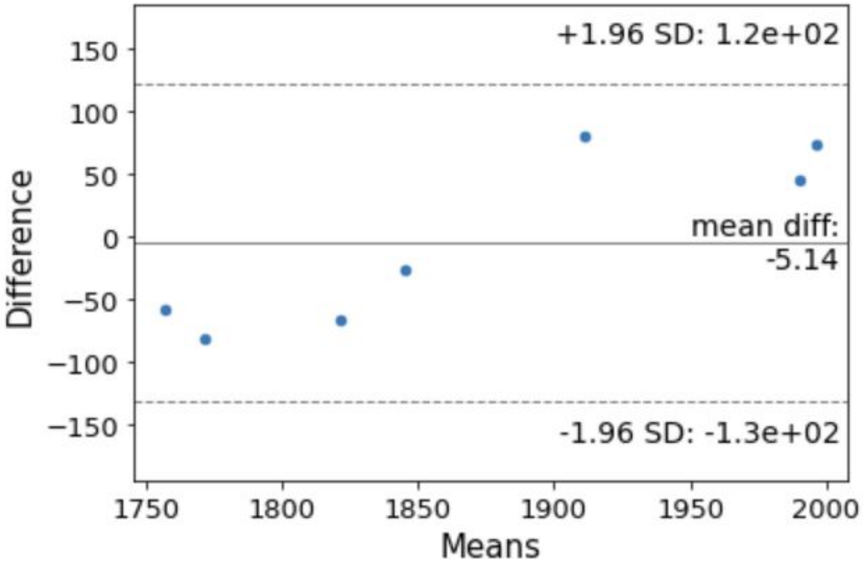

Fig. S5. Bland-Altman plot showing robustness in acquisition in 7 cases across gestational age.
